# Editorial: Scene-dependent image quality and visual assessment

**DOI:** 10.3389/fnins.2023.1205341

**Published:** 2023-05-10

**Authors:** John Jarvis, Sophie Triantaphillidou, Mylene Farias, Robin Jenkin

**Affiliations:** ^1^Department of Computer Science and Engineering, University of Westminster, London, United Kingdom; ^2^Department of Computer Science, Texas State University, San Marcos, TX, United States; ^3^NVIDIA, Santa Clara, CA, United States

**Keywords:** image quality, visual modeling, visual assessment, scene and process dependency, imaging system performance, image quality metrics

Image quality is defined as the degree of excellence the image conveys. It is assessed using visual (image) psychophysics and predictive modeling. Engineering type image quality models, employed in imaging system design and optimization, are based on the assumption that the perceived image quality is a function of both the imaging system performance and the human visual system performance. Such models employ imaging system performance parameters related to color, sharpness, resolution, noise, etc. as input parameters, along with models of the human visual system. System performance parameters are typically derived from simple test charts depicting well-characterized signals captured under strict laboratory conditions. Such signals include, for example, uniform color patches with known lightness and chroma, sinusoidal exposures, edges and random noise with known spatial frequency contents and contrast. Similarly, vision models employed in image quality modeling are largely based on visual measurements obtained from simple test stimuli, such as sine-waves, Gabor functions, random noise, and uniform color patches. The modeled image quality is, as a result, system-dependent but scene content-independent. Computational models have also been used in the determination of image quality and these base their predictions on the contents of captured and processed images. The modeled image quality, in these cases, is scene content dependent, but the system performance parameters are undifferentiated.

There are many unresolved challenges when it comes to the assessment of image and video quality. To begin with, engineering-type quality models that account for imaging system performance, fail to account for scene content, since they do not address the action of advanced algorithms which are incorporated into commercial imaging systems. These algorithms are image content aware and become more or less active depending on the individual image structures, local tones and colors. In contrast, computational quality models derived directly from images of individual natural scenes fail to directly relate to component parameters in the imaging system hardware and signal image processes. They are therefore unfit for system design and optimization.

Recently, advances have been made in measuring and modeling human spatial vision, as well as spatial camera performance directly from natural scene images. In human vision studies, contrast detection and discrimination sensitivity (directly relating to perceived sharpness and resolution) were determined from the images' narrow spatial frequency bands, viewed within the context of the complete image contents (Triantaphillidou et al., 2019; Jarvis et al., 2022). Therefore, they provide visual system performance estimates which are scene content dependent. In camera measurement studies, scene-and-process-dependent camera Modulation Transfer Functions and the Noise Power Spectra were derived from printed natural images (Fry et al., 2019a), and more recently directly from natural scene captures (van Zwanenberg et al., 2021a,b). Initial research indicated that such scene content- dependent visual and camera functions were of benefit to “purer in form” quality metrics that do not rely on observers' data fittings (Fry et al., 2019b).

Nonetheless, studies such as those mentioned above are only just starting to appear in the literature, and there are still many more questions to answer. With respect to the human observer, how do psychological factors such as aesthetic appreciation, individual preferences and attention span impact quality? Cultural differences may also impact quality estimations, particularly with respect to preference for color hue and saturation. Further research on scene- and process- dependent image quality assessment is also necessary in the evaluation of the robustness of non-human (machine) perception systems; such research is still in its infancy.

The six articles presented in this Research Topic address a wide range of challenges in assessing image quality, embracing topics relating to color perception, human decision-making processes, aesthetics, the impact of optical glare and the application of neural network theory.

On the subject of aesthetics, Leder et al. present a taxonomy of factors likely to contribute to image quality and perceived beauty in mobile phone images. A model for visual processing is described, which addresses important psychological factors that are not incorporated in traditional quality models (which focus mainly on image properties such as resolution and pictorial noise). The model is an extension of a previous version describing aesthetic appreciation in art and could offer an important future framework for our understanding of quality and beauty in reproduced images in general.

Further psychological factors are covered in a study of the decision–making heuristics involved in quality estimations outlined by Leisti et al.. The authors present three experiments designed to investigate psychological factors underlying quality appraisal and the length of time taken in the process. The results obtained highlight a number of important factors to be taken into consideration when experimentally measuring human image quality preferences.

Umair Arif et al. investigate the perception of 3D virtual images appearing within a real visual environment using a reaction time experiment. The work provides some interesting results and quantifies the accuracy of the reaction time data by measuring and analyzing omitted, completed and anticipated responses. The development of commercial products employing 3D objects immersed within an image, is expanding and the results presented by the authors will provide useful background in the evolution of this technology.

Color science features in the contribution from Xie et al. who examine the concept that a larger display color gamut volume (CGV) produces higher perceived brightness and chroma. In this study, RGBW (RGB plus white channel) displays were investigated in a paired comparison experiment to obtain scales of color gamut expressed as perceived brightness and colorfulness. The results show that the actual structure of a reproduced image is important when optimizing CGV.

Artificial neural networks form the basis of a study by Prabhushankar and AlRegib who outline a thorough review of problems associated with deep neural network models when noise is introduced either from the imaging stages or from environmental factors. A new concept termed “stochastic surprisal” is introduced, which relates to the network and the input and this is tested through image quality assessments and recognition.

Finally, the visual mechanism relating to glare as a component in image quality modeling is outlined by McCann et al.. The authors give a comprehensive review of previous work on glare and its impact on brightness perception and provide a computational model of glare, with potential to provide a useful component for incorporation in future image quality models.

## Author contributions

All authors listed have made a substantial, direct, and intellectual contribution to the work and approved it for publication.
